# Genomic diversity of *Vibrio* spp. and metagenomic analysis of pathogens in Florida Gulf coastal waters following Hurricane Ian

**DOI:** 10.1128/mbio.01476-23

**Published:** 2023-10-16

**Authors:** Kyle D. Brumfield, Moiz Usmani, Sanneri Santiago, Komalpreet Singh, Mayank Gangwar, Nur A. Hasan, Michael Netherland, Katherine Deliz, Christine Angelini, Norman L. Beatty, Anwar Huq, Antarpreet S. Jutla, Rita R. Colwell

**Affiliations:** 1 Maryland Pathogen Research Institute, University of Maryland, College Park, Maryland, USA; 2 University of Maryland Institute for Advanced Computer Studies, University of Maryland, College Park, Maryland, USA; 3 Department of Environmental Engineering Sciences, Geohealth and Hydrology Laboratory, University of Florida, Gainesville, Florida, USA; 4 Department of Environmental Engineering Sciences, Engineering School of Sustainable Infrastructure and Environment, University of Florida, Gainesville, Florida, USA; 5 EzBiome Inc., Gaithersburg, Maryland, USA; 6 Department of Medicine, Division of Infectious Diseases and Global Medicine, University of Florida, Gainesville, Florida, USA; University of Nebraska-Lincoln, Lincoln, Nebraska, USA

**Keywords:** *Vibrio vulnificus*, *Vibrio parahaemolyticus*, *Vibrio*, climate change, hurricane, whole-genome sequencing, metagenomics, antimicrobial resistance, virulence, pathogens, predictive intelligence

## Abstract

Changing climatic conditions influence parameters associated with the growth of pathogenic *Vibrio* spp. in the environment and, hence, are linked to increased incidence of vibriosis. Between 1992 and 2022, a long-term increase in *Vibrio* spp. infections was reported in Florida, USA. Furthermore, a spike in *Vibrio* spp. infections was reported post Hurricane Ian, a category five storm that made landfall in Florida on 28 September 2022. During October 2022, water and oyster samples were collected from three stations in Lee County in an area significantly impacted by Ian. *Vibrio* spp. were isolated, and whole-genome sequencing and phylogenetic analysis were done, with a focus on *Vibrio parahaemolyticus* and *Vibrio vulnificus* to provide genetic insight into pathogenic strains circulating in the environment. Metagenomic analysis of water samples provided insight with respect to human health-related factors, notably the detection of approximately 12 pathogenic *Vibrio* spp., virulence and antibiotic resistance genes, and mobile genetic elements, including the SXT/R391 family of integrative conjugative elements. Environmental parameters were monitored as part of a long-term time series analysis done using satellite remote sensing. In addition to anomalous rainfall and storm surge, changes in sea surface temperature and chlorophyll concentration during and after Ian favored the growth of *Vibrio* spp. In conclusion, genetic analysis coupled with environmental data and remote sensing provides useful public health information and, hence, constitute a valuable tool to proactively detect and characterize environmental pathogens, notably vibrios. These data can aid the development of early warning systems by yielding a larger source of information for public health during climate change.

**IMPORTANCE:**

Evidence suggests warming temperatures are associated with the spread of potentially pathogenic *Vibrio* spp. and the emergence of human disease globally. Following Hurricane Ian, the State of Florida reported a sharp increase in the number of reported *Vibrio* spp. infections and deaths. Hence, monitoring of pathogens, including vibrios, and environmental parameters influencing their occurrence is critical to public health. Here, DNA sequencing was used to investigate the genomic diversity of *Vibrio parahaemolyticus* and *Vibrio vulnificus*, both potential human pathogens, in Florida coastal waters post Hurricane Ian, in October 2022. Additionally, the microbial community of water samples was profiled to detect the presence of *Vibrio* spp. and other microorganisms (bacteria, fungi, protists, and viruses) present in the samples. Long-term environmental data analysis showed changes in environmental parameters during and after Ian were optimal for the growth of *Vibrio* spp. and related pathogens. Collectively, results will be used to develop predictive risk models during climate change.

## INTRODUCTION

Climate change is reported to be associated with an increased frequency of anomalous weather events, notably heatwaves and severe precipitation which can significantly impact the marine environment, especially along the coast (1). Environmental factors, such as temperature, salinity, chlorophyll, and sea surface height, can influence the incidence and transmission of pathogenic agents by impacting the proliferation, dissemination, and virulence of microorganisms in the environment. Climatic conditions also have the potential to influence human behavior by more frequent contact with pathogens through water-related activities during periods of warming (2, 3). Hence, climate change associated with the shifts in the geographical range of microbial species has the potential to influence the emergence and re-emergence of disease (4). A dramatic example is the significant geographic expansion of pathogenic *Vibrio* spp., a finding corroborated by their impact on public health, namely, increased numbers of reported vibriosis infections in humans, as well as aquaculture loss (5–13).

*Vibrio* spp. are autochthonous to the aquatic environment, notably in high concentrations along the coast and their incidence is strongly influenced by environmental conditions (10, 11). *Vibrio* spp. play an important role in the degradation of polymeric substances, such as chitin, and in other significant biogeochemical processes (10, 14, 15). *Vibrio* spp. occur in aquatic ecosystems as free-living single cells or in aggregates, e.g., within biofilms in high numbers and attached to various abiotic substrates. They have been shown to be commensals and symbionts of aquatic invertebrates, such as crustaceans, zooplankton, and bivalves, all of which are known to host these bacteria (10, 16–22). Copepods, zooplankton comprising a significant component of aquatic fauna, are a major host of *Vibrio* spp. and are considered a vector of *Vibrio cholerae* (5, 18, 19, 23, 24). *Vibrio* spp. concentrate in filter-feeding shellfish, especially oysters, which are often consumed raw thereby exposing people to large doses of potentially pathogenic agents (16, 25, 26). Furthermore, *Vibrio* spp. with bioluminescent properties are important symbionts of marine organisms, including *Vibrio fischeri* which colonizes the light-emitting organ of the Hawaiian bobtail squid, *Euprymna scolopes* (27), and luminescent *V. cholerae* and *Vibrio vulnificus* strains reported to be associated with copepods and related crustacean species (18, 20–22, 28).

Several species of the genus *Vibrio* cause severe infection in humans, primarily related to consumption of contaminated seafood or exposure to water containing the pathogens (29). *V. cholerae* is well documented as the etiological agent of cholera, the seventh cholera pandemic of which is in progress and continues to plague the modern world, notably when climate/weather processes, microbiological parameters, and sociological determinants intersect with population vulnerabilities and loss of water, sanitation, and hygiene infrastructure (30–32). In addition to *V. cholerae*, *Vibrio parahaemolyticus* and *V. vulnificus* have proven historically significant (10, 29). *V. parahaemolyticus*, first described by Professor Fujino during a 1950 shirasu food poisoning outbreak in Japan (33), is a major cause of seafood-derived foodborne gastroenteritis in the United States (34). *V. vulnificus*, first reported in the U.S. in 1976 (35), is also a common cause of foodborne illness and can cause severe extraintestinal infections, including necrotizing fasciitis and septicemia (36). The bacterium has a fatality rate that is one of the highest of any waterborne pathogen, i.e., greater than 50% for primary septicemia. It also is responsible for ca. 95% of all waterborne and seafood-derived foodborne deaths in the United States (36, 37). In the United States, *Vibrio* spp. are estimated to cause ca. 80,000 illnesses and hundreds of deaths annually, of which ca. 65% are foodborne (34, 38). According to the Centers for Disease Control and Prevention FoodNet, which has sites in 10 states covering 15% of the U.S. population, there is indication of a long-term increase in reported vibriosis between 1996 and 2019 (10, 39). In the eastern United States between 1988 and 2018, *V. vulnificus* wound infections increased eightfold, i.e., from 10 to 80 cases per annum, and the northern case limit has shifted northward geographically at ca. 48 km per annum (13). Environmental factors linked to climate change have the potential to enhance the incidence and genomic diversity of these pathogens in the environment, especially in coastal communities, and this trend of increased vibriosis is expected to continue (5, 40).

A key issue related to the risk of vibriosis outbreaks is the high population density and economic activity along coastal areas, and the U.S. eastern seaboard is a prime example. In fact, ca. 40% of the total U.S. population lives in coastal communities, with nearly half in an elevated health risk category, e.g., elderly and/or low-income households (41). The state of Florida (FL) has the longest coastline of any of the 48 contiguous states in the United States, and ca. 22% of the population of FL is over the age of 65 (42). Furthermore, many FL residents rely on protein from seafood, especially aquaculture. While FL does have distinct seasons, i.e., warmest waters in the summer and early fall, the climate is generally warm year-round which allows for increased duration and frequency of recreational and occupational activities, along with an extended seasonality of *Vibrio* spp. abundance.

Pathogenic *Vibrio* spp. that cause cholera and vibriosis have been reportable diseases in FL since 1981 (34). Between 2004 and 2021, over 3,000 vibriosis cases and 205 deaths were reported in FL (43). *V. parahaemolyticus* and *V. vulnificus* accounted for over ca. 40% of the vibriosis cases and have been of particular interest to the State because of their potential association with locally harvested shellfish (43). Between 2000 and 2021, there has been an average of ca. 34 (ranging from 13 to 55) *V. parahaemolyticus* and ca. 35 (ranging from 15 to 51) *V. vulnificus* cases reported each year, with most infections occurring between May and October (43). However, it is worth noting that *V. vulnificus* infections accounted for ca. 78% of the confirmed deaths associated with vibriosis during this period.

On 28 September 2022, Hurricane Ian, a destructive category five storm, made landfall in southwestern FL, bringing anomalously heavy rainfall, high winds (ca. 240 km/h), and dangerous surf to the area, resulting in a rise in seawater levels, flash flooding, and mass destruction of infrastructure around coastal areas. In the days following Hurricane Ian, many FL residents were impacted by sustained floodwaters, a problematic situation given that pathogenic *Vibrio* spp., namely, *V. vulnificus* and *V. parahaemolyticus*, thrive in warm and low-salinity waters (10, 11, 44). According to the FL Department of Health (DOH), there were 74 reported cases of *V. vulnificus* infections, with 17 confirmed deaths in 2022 (43). Furthermore, 38 of the cases and 11 vibriosis-associated deaths were attributed to the storm, accounting for nearly double the usual number for that time of year (45, 46). Hence, routine monitoring and predictive intelligence models warning decision-makers and individuals when risk of infection of *Vibrio* spp. (and other pathogens) is high are essential to safeguarding public health.

Here, we describe whole-genome sequencing (WGS) analysis to characterize pathogenic *Vibrio* spp. isolates (*V. vulnificus* and *V. parahaemolyticus*) recovered from water and oyster samples along the FL Gulf Coast (FGC) in October 2022. We also profiled water samples, employing shotgun metagenomic sequencing (SMS), for the detection of *Vibrio* spp. and related pathogens, along with their virulence factors (VFs), antimicrobial resistance genes (ARGs), and mobile genetic elements (MGEs). These results provide useful information for future investigations, both for inhabitants of the FGC and globally, to evaluate the incidence of pathogenic *Vibrio* populations relative to climate change over time.

## RESULTS

### Anomalous weather events associated with Hurricane Ian

**Fig 1:**
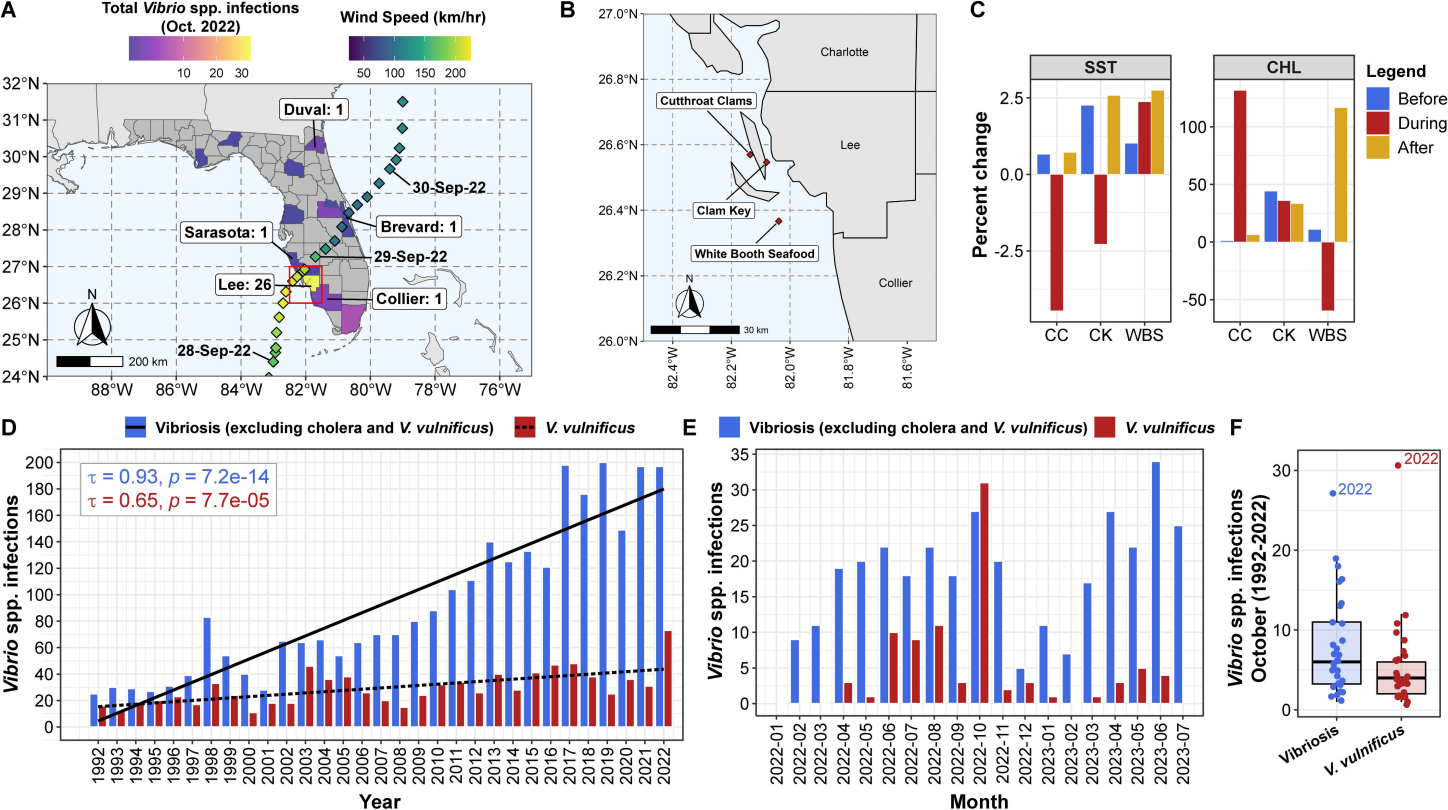
Area of study. (**A**) Path of Hurricane Ian and total number of confirmed *Vibrio* spp. infections in FL at the county level for October 2022 (43, 45). Counties reporting *V. vulnificus* cases and number are indicated. Track, timeline, and windspeed of Hurricane Ian, indicated by diamonds, were retrieved from the International Best Track Archive for Stewardship (IBTrACS) project (47). The outlined red box indicates an area of sampling locations. (**B**) Map of sampling locations, indicated by red diamonds. Scale bar corresponds to distance according to World Map Data from Natural Earth (48). (**C**) Percent change in a time series analysis to determine potential impact of anomalous weather events before (24 July 2022 to 23 September 2022), during (24 September 2022 to 30 September 2022), and after (1 October 2022 to 30 November 2022) Hurricane Ian, for sea surface temperature (SST) and chlorophyll (CHL). (**D**) Confirmed vibriosis (excluding cholera and *V. vulnificus*) and *V. vulnificus* cases between 1992 and 2022. Correlations among axis variables were respectively generated using Kendall’s tau method. (**E**) Confirmed vibriosis (excluding cholera and *V. vulnificus*) and *V. vulnificus* cases at a monthly scale between January 2022 and July 2023. (**F**) Confirmed vibriosis (excluding cholera and *V. vulnificus*) and *V. vulnificus* cases during the month of October between 1992 and 2022. Boxes summarize distribution by indication of interquartile range (IQR), with median shown as the center bar of each group. Whiskers represent 1.5 times the IQR.

Hurricane Ian made landfall on 28 September 2022, just south of Charlotte County near Pirate Harbor, with maximum sustained winds of 240 km/h (Fig. 1A). On 26 October 2022, samples were collected from three stations adjacent to this area, including Cutthroat Clams (CC), Clam Key (CK), and White Booth Seafood (WBS) (Fig. 1B). Nomenclature of the sampling stations is representative of station proximity to aquaculture facilities, and it should be noted that samples were not collected directly from the named facilities in this study. Environmental parameters recorded at the time of sample collection are detailed in Table 1. Overall, water temperature, pH, and optical dissolved oxygen (DO) were similar across the three stations. However, salinity was higher at CC (30 PPT), compared to the CK and WBS stations (ca. 20 PPT). Following long-term time series analysis (Fig. 1C), it was observed that all stations experienced above-average sea surface temperature (SST) before Hurricane Ian, varying between 0.6% and 2.26%, within half of the standard deviation (SD) compared to the previous 10 years. During Hurricane Ian (25–30 September 2022), CC and CK experienced a decrease in SST. Following Ian (1 October to 30 November 2022), all stations showed greater than average SST, varying between 0.7% and 2.75%. With respect to chlorophyll, all sampling sites indicated above-average chlorophyll concentration before the hurricane, varying between 1.3% and 45%. Notably, the CK station indicated significant chlorophyll variability, greater than one SD compared to the previous 10 years. However, during and after Ian, these locations experienced positive anomalous chlorophyll with values as high as five times the SD.

**TABLE 1:** Environmental parameters recorded during sample collection on 26 October 2022

Parameter	Cutthroat Clams^*a*^	Clam Key^*a*^	White Booth Seafood^*a*^
Water temp (°C)	25.4/24.8 (25.1)	24.6/24.4 (24.5)	24.5/24.4 (24.45)
pH	8.00/8.07 (8.035)	7.83/7.85 (7.84)	7.79/7.90 (7.845)
DO (mg/L)	6.0/6.1 (6.05)	6.2/5.3 (5.75)	6.1/5.2 (5.65)
Salinity (PPT)	30.36/30.60 (30.48)	19.74/19.75 (19.745)	19.84/19.84 (19.84)

^*a*^ Environmental parameters presented as surface recording/bottom recording (mean).

### *V. vulnificus* clinical reports

The incidence of vibriosis, excluding cholera and *V. vulnificus* (Tau = 0.93, *P* < 0.0001), and *V. vulnificus* (Tau = 0.65, *P* < 0.0001) infections for the State of FL increased significantly from 1992 to 2022 (Fig. 1D). In 2022, the total number of confirmed *V. vulnificus* cases, 74, and deaths, 17, was roughly double that of 2021 (cases = 34; deaths = 10) and 2020 (cases = 36; deaths = 7). Fig. 1E shows the number of vibriosis and *V. vulnificus* infections per month between January 2022 and July 2023. Notably, a spike in *V. vulnificus* cases was observed in October 2022. Furthermore, the number of vibriosis and *V. vulnificus* infections reported during October 2022 represents the highest number of cases on record for the month since 1992 (Fig. 1F). In October 2022, the highest number of vibriosis, eight, and *V. vulnificus*, 26, cases was observed in Lee County, an area significantly impacted by Hurricane Ian (Fig. 1A). Similarly, the greatest number of *V. vulnificus* deaths was reported in Lee County, which reported eight deaths in 2022. Other areas reporting *V. vulnificus* deaths in 2022, including Escambia, Bay, Citrus, Broward, Highlands, Brevard, and Seminole, were located along the coast, except for Polk County which had been in the path of Ian. However, it is worth noting that the number of reported *V. vulnificus* deaths did not increase significantly in FL from 2008 to 2022 (Tau <0.3, *P* > 0.05).

### PCR detection of *Vibrio* spp.

Detection of genetic markers for the genus *Vibrio* (16S rRNA), *V. parahaemolyticus* (*tlh*, *tdh*, and *trh*), *V. vulnificus* (*vvhA*), and *V. cholerae* (*rfb*-O1, *rfb*-O139, and *ctxA*) was done for water and oyster samples (Table 2). The *Vibrio* spp. 16S rRNA marker was detected in all alkaline peptone water (APW) enriched samples (water and oyster) from each location. However, successful detection of *tlh* and/or *vvhA* was possible only with APW enrichment, with PCR analysis of DNA done directly on oyster homogenate and Sterivex filters negative in all samples. Specifically, the *tlh* marker was detected in enriched water samples at all locations and APW-enriched oyster samples collected at CK. However, *V. parahaemolyticus* primary VFs (*tdh* and *trh*) were not detected. With respect to *V. vulnificus*, the *vvhA* marker was detected in CK and WBS enriched water samples but not in oyster samples. Lastly, markers coding for toxigenic *V. cholerae* (*ctxA*, *rfb*-O1, and *rfb*-O139) were not detected.

**TABLE 2:** Primers, PCR parameters, and reference strains used in this study

Description (reference)	Oligonucleotide name	Sequence (5’−3’)	PCR product size (bp)	Annealing temp (°C)	Reference strain used in this study
*Vibrio* genus 16S rRNA (49)	567F	GGCGTAAAGCGCATGCAGGT	120	55	*V. cholerae* ATCC 39315^*a*^
	680R	GAAATTCTACCCCCCTCTACAG			
*toxR* of *V*. *parahaemolyticus* (Vp), *V*. *cholerae* (Vc), and *V*. *vulnificus* (Vv) (50)	UtoxF	GASTTTGTTTGGCGYGARCAAGGTT			
	vptoxR	GGTTCAACGATTGCGTCAGAAG	297 (*Vp*)	60	*V*. *parahaemolyticus* ATCC 17803
	vctoxR	GGTTAGCAACGATGCGTAAG	640 (*Vc*)	55	*V*. *cholerae* ATCC 39315
	vvtoxR	AACGGAACTTAGACTCCGAC	435 (*Vv*)	55	*V*. *vulnificus* ATCC 27562
Total and hemolysin-producing *V*. *parahaemolyticus via tlh*, *tdh*, and *trh* (51)	L-tl	AAAGCGGATTATGCAGAAGCACTG	450 (*tlh*)	58	*V*. *parahaemolyticus* ATCC 17803
	R-tl	GCTACTTTCTAGCATTTTCTCTGC			
	L-tdh	GTAAAGGTCTCTGACTTTTGGAC	269 (*tdh*)	58	*V*. *parahaemolyticus* TX2103 (*tdh*+/*trh*−)^*b*^
	R-tdh	TGGAATAGAACCTTCATCTTCACC			
	L-trh	TTGGCTTCGATATTTTCAGTA	500 (*trh*)	58	*V*. *parahaemolyticus* AQ4037 (*tdh*−/*trh+*)^*b*^
	R-trh	CATAACAAACATATGCCCATTTCCG			
*V*. *vulnificus* hemolysin *vvhA* (52)	L-vvh	TTCCAACTTCAAACCGAACTATGAC	205	58	*V*. *vulnificus* ATCC 27562
	R-vvh	ATTCCAGTCGATGCGAATACGTTG			
Toxigenic *V*. *cholerae* O1 and O139 via *ctxA*, *rfb-*O1, and *rfb*-O139 (53)	O139F2	AGCCTCTTTATTACGGGTGG	449 (O139)	55	*V*. *cholerae* MO10 (O139)^*c*^
	O139R2	GTCAAACCCGATCGTAAAGG			
	O1F2	GTTTCACTGAACAGATGGG	192 (O1)	55	*V*. *cholerae* ATCC 39315^*a*^
	O1R2-2	GGTCATCTGTAAGTACAAC			
	VCT1	ACAGAGTGAGTACTTTGACC	308 (*ctxA*)	55	*V*. *cholerae* ATCC 39315^*a*^
	O139F2	AGCCTCTTTATTACGGGTGG	449 (O139)	55	*V*. *cholerae* MO10 (O139)^*c*^

^*a*^ Isolate synonym of N16961.

^*b*^ Isolate characterized previously (54).

^*c*^ Isolate characterized previously (55).

### PCR characterization of *Vibrio* spp.

Following APW enrichment and subculture plating on selective media (ChromAgar, TCBS, and VVA), a total of 21 presumptive *Vibrio* spp. were isolated from the water (*n* = 14) and oyster samples (*n* = 7). All isolates were identified as members of the genus *Vibrio*, determined by PCR for presence of the *Vibrio* spp. 16S rRNA marker. Nine isolates were positive for *V. parahaemolyticus toxR* and *tlh*, and all were negative for *tdh* and *trh*. Similarly, 12 isolates were positive for *V. vulnificus toxR* and *vvhA*. All isolates were negative for *V. cholerae toxR* and toxigenic markers (*rfb*-O1, *rfb*-O139, and *ctxA*).

### Comparative genomics of *Vibrio* spp. isolates employing WGS

**Fig 2:**
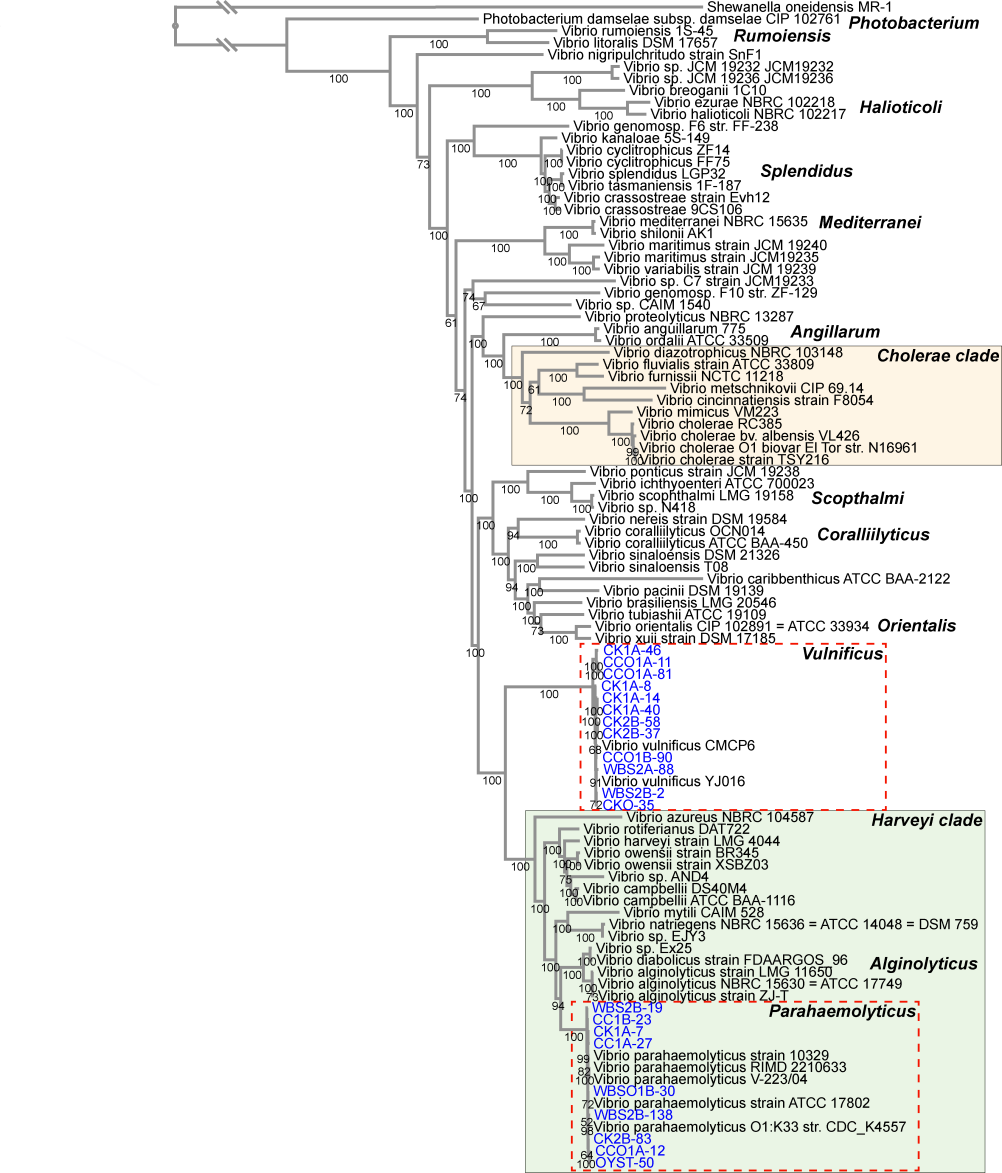
Phylogenetic relatedness of *Vibrionaceae* spp. Codon tree was created using 344 coding sequences (ca. 7.13 × 10^4^ amino acids in length) shared by all genomes with the LG model. *Shewanella oneidensis* MR-1 was used as an outgroup to root the tree. Bootstrap values are shown as percentages. *Vibrio* spp. isolates collected during this study are shown in blue. Trees were built using the Bacterial and Viral Bioinformatics Resource Center (BV-BRC) software package (56) and Randomized Axelerated Maximum Likelihood (RAxML) algorithm automatic model selection (57).

The 21 presumptive *Vibrio* spp. isolates were subjected to WGS and comparative genomics. Following annotation, phylogenetic trees were built from the core genome, determined by the alignment of coding sequences from selected isolates included in the analysis to evaluate genetic relatedness of the *Vibrio* spp. isolated in this study to reference genomes previously reported (Fig. 2). A search for homologous genes returned 344 coding sequences (ca. 7.13 × 10^4^ amino acids in length) shared by all genomes. The best-fit protein model found by RAxML was Le and Gascuel (LG). Within the *Vibrionaceae* phylogeny, each species formed coherent clusters in taxonomic subclades. Notably, the *V. cholerae* clade comprised *V. cholerae*, *Vibrio mimicus*, *Vibrio cincinnatiensis*, *Vibrio metschnikovii*, *Vibrio furnissii*, *Vibrio fluvialis*, and *Vibrio diazotrophicus*. The 12 suspected *V. vulnificus* isolates obtained in this study formed a distinct cluster with reference *V. vulnificus* strains. Similarly, nine presumptive *V. parahaemolyticus* isolates were clustered with reference *V. parahaemolyticus* strains. All *V. parahaemolyticus* strains were placed in a subclade within the *Harveyi* clade that also included *Vibrio alginolyticus*, *Vibrio diabolicus*, *Vibrio campbellii*, *Vibrio owensii*, and *Vibrio harveyi*.

**Fig 3:**
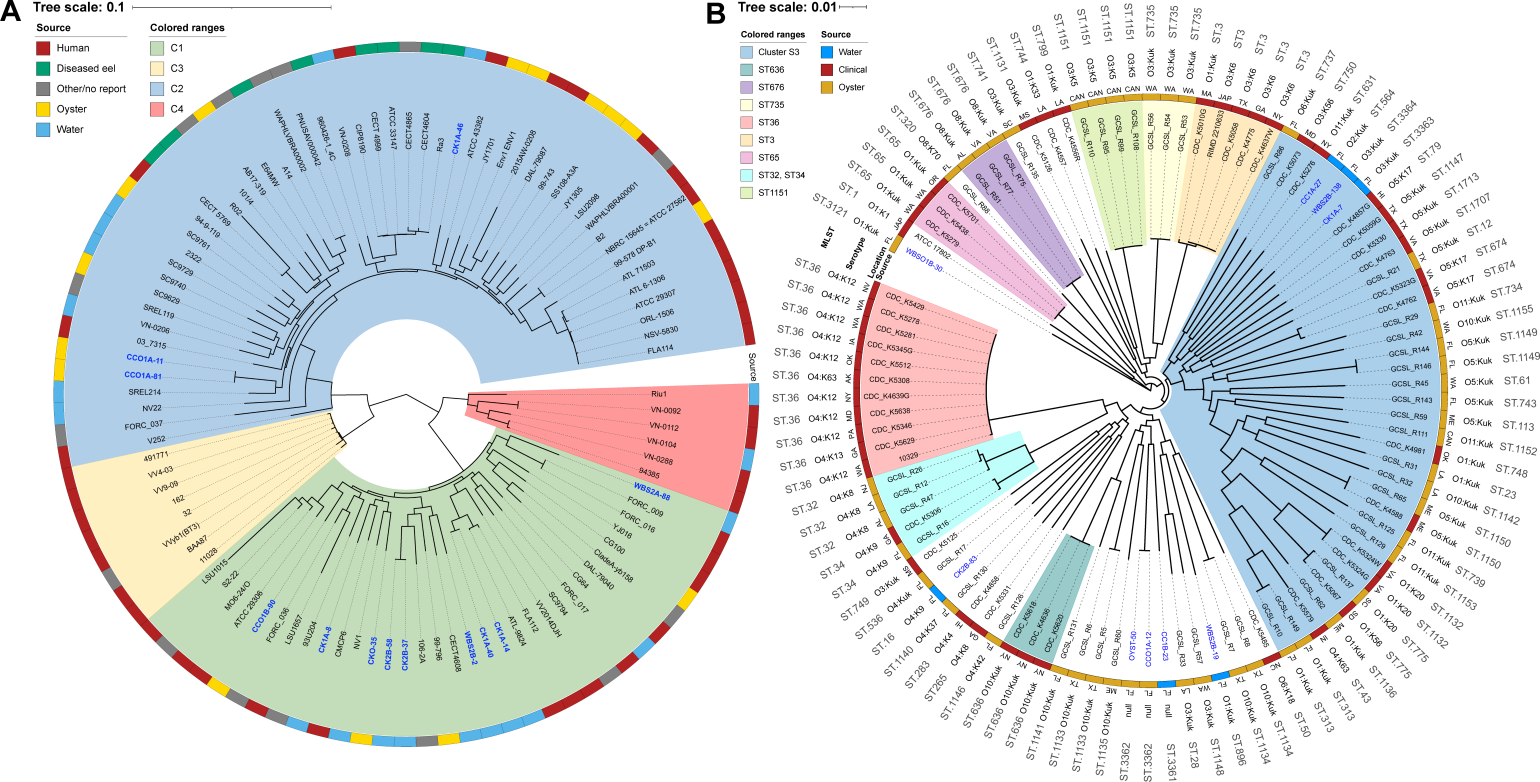
Phylogenetic relationships of *Vibrio vulnificus* and *Vibrio parahaemolyticus*. (**A**) *V. vulnificus* codon tree was created using 982 coding sequences (ca. 3.02 × 10^5^ amino acids in length) shared by all genomes with the JTT model. (**B**) *V. parahaemolyticus* codon tree was created using 1,000 coding sequences (ca. 3.88 × 10^5^ amino acids in length) shared by all genomes with the HIVB model. *Vibrio* spp. isolates collected during this study are shown in blue. Trees were built using the Bacterial and Viral Bioinformatics Resource Center (BV-BRC) software package (56) and Randomized Axelerated Maximum Likelihood (RAxML) algorithm automatic model selection (57).

Nearest neighbors of isolates from this study were identified using core-genome phylogeny in the context of major clonal lineages for *V. vulnificus* (Fig. 3A) and *V. parahaemolyticus* (Fig. 3B). Homologous gene search returned 1,000 coding sequences, the maximum number allowed by the software program for *V. parahaemolyticus* (ca. 3.88 × 10^5^ amino acids; best-fit model: Human Immunodeficiency Virus-Between [HIVB]) and 982 for *V. vulnificus* (ca. 3.02 × 10^5^ amino acids; best-fit model: Jones-Taylor-Thornton [JTT]).

### Phylogenetic relatedness of *V. vulnificus* isolates

The 12 *V*. *vulnificus* isolates (four from oyster and eight from water) were assigned to eight multilocus sequence type (MLST) profiles (using alleles for *glp*, *gyrB*, *mdh*, *metG*, *purM*, *dtdS*, *lysA*, *pntA*, *pyrC*, and *tnaA*), including ST621–ST628 (Table S4). Core genome phylogeny was used to investigate genomic relatedness of *V. vulnificus* isolates from this study with established lineages, representing isolates recovered from a wide range of geographical and ecological sources (58). All strains clustered into four distinct groups (C1 to C4). C1 and C2 are significantly divergent lineages including much of the clinical isolate diversity. By comparison, C3 and C4 indicate high clonality. Nine isolates were phylogenetically joined in C1 and three in C2. However, there was no distinct clustering pattern linking sample location, source of isolation, or virulence.

### Phylogenetic relatedness of *V. parahaemolyticus* isolates

The nine *V. parahaemolyticus* isolates (three from oyster and six from water) were assigned to eight MLST profiles (using alleles for *dnaE*, *gyrB*, *recA*, *dtdS*, *pntA*, *pyrC*, and *tnaA*), including ST16, ST564, ST896, ST3121, and ST3361-ST3364, and one isolate was assigned to clonal complex 49 (Fig. 3B; Table S5). Based on the core genome phylogeny of *V. parahaemolyticus* isolates from this study and established phylogenetic and biogeographic patterns of strains from North America (59), distinct population structures were observed, with at least nine well-supported clades. Four clades (ST36, ST636, ST65, and ST3) comprised clinical sources. Three clades (ST676, ST1151, and ST735) were from oysters, and the remaining (cluster S3 and ST32/ST34) were of both clinical and environmental origin. Four isolates (OYST-50, CCO1A-12, CC1B-23, and WBS2B-19) composed a clade of primarily environmental isolates. Like *V. vulnificus* phylogeny, no distinct clustering pattern was observed linking *V. parahaemolyticus* phylotype from FGC.

### Genetic characterization of the *Vibrio* spp.

**Fig 4:**
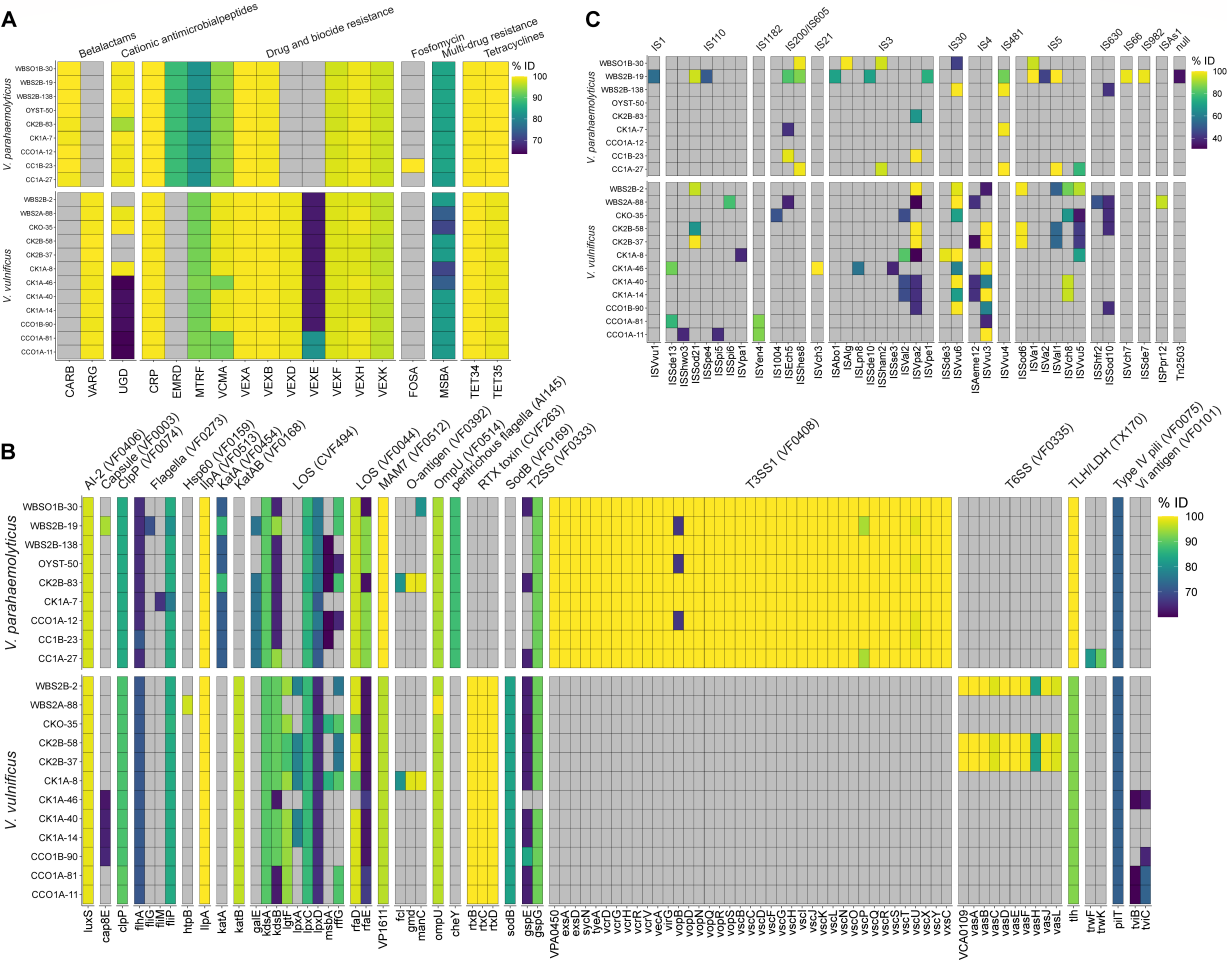
Genomic characterization of *Vibrio* spp. isolates. Assembled contigs were characterized to determine carriage of (**A**) antimicrobial drug, biocide, and metal resistance determinants using the Microbial Ecology Group Antimicrobial Resistance (MEGARes) database (60), (**B**) virulence factors the Virulence Factor Database (VFDB) (61), and (**C**) MGEs the Mobile Genetic Element database (MGEdb) (62), and ABRicate (63) with identity and coverage thresholds set to 60%.

All isolates examined in this study carried homologous genes coding for ARG, VFs, and MGEs (Fig. 4). Overall, the *Vibrio* spp. showed similar patterns of resistance, with minor variation in multi-drug and biocide resistance, namely, *msba*, the *vex* operon (*vexA*, *vexB*, *vexD*, *vexE*, *vexF, vexH*, and *vexK*), and tetracycline resistance encoded by *tet(34)* and *tet(35)*. Interestingly, *V. parahaemolyticus* isolates carried *carB* while *V. vulnificus* carried *varG*, both coding resistance to beta-lactams. One isolate, CC1B-23, carried genes associated with fosfomycin resistance.

Many VF homologs were detected in the *Vibrio* spp. isolates, namely, genes coding for outer membrane adhesion factors, flagella, stress response, and biofilm formation. Type 3 secretion system (T3SS) (type 1) and TLH/LDH were detected in all *V. parahaemolyticus* strains, while the type 6 secretion system (T6SS) and RTX toxin (Repeats in ToXin) were detected in *V. vulnificus*. Major integrative conjugative elements (ICEs) encoding VFs and ARGs were not detected, but several insertion sequences (ISs), transposase genes flanked by two inverted repeats, were detected. Overall, insertion sequences were more common in strains recovered from water, compared to those from oysters. IS3 and IS5 insertion sequence families were common in both *V. parahaemolyticus* and *V. vulnificus*, while the IS4 family of insertion sequences were detected only in *V. vulnificus*.

### Metagenomic data analysis

#### Microbiome community profile

**Fig 5:**
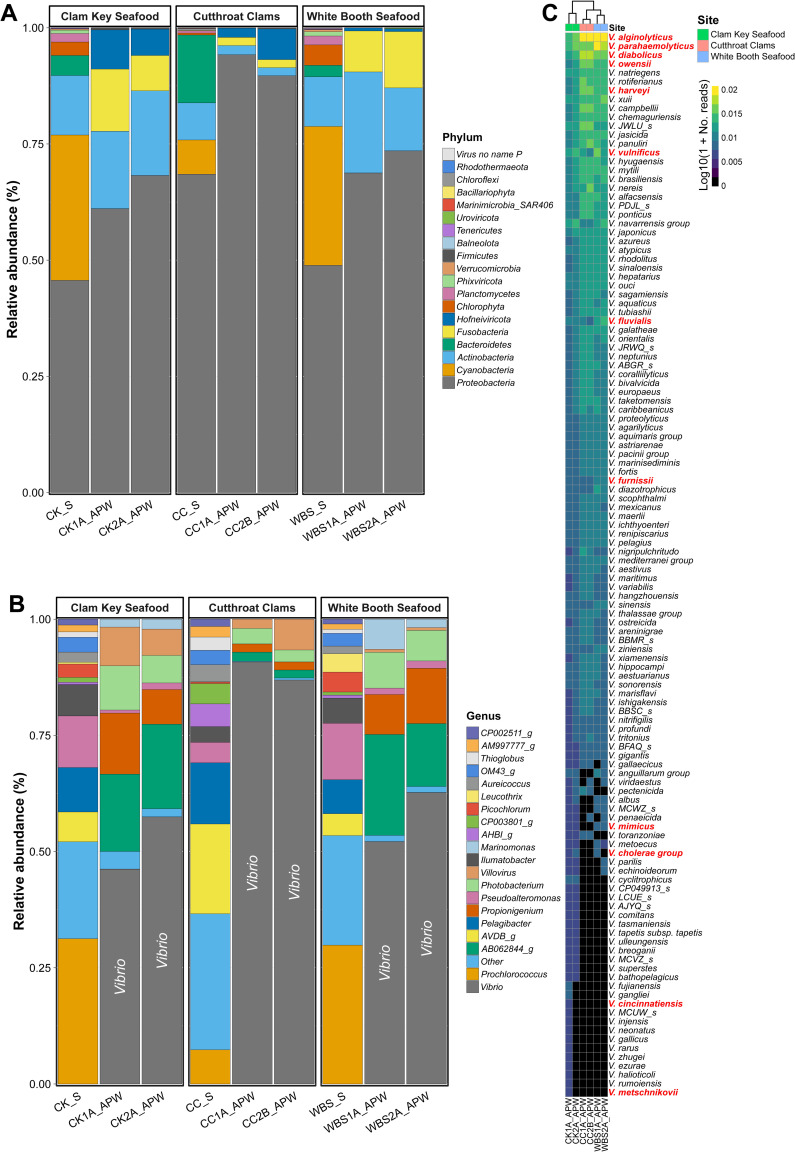
Microbial community composition. Metagenomic community profiling was done using up-to-date bacterial core gene (UBCG) algorithm (64) with EzBioCloud database (65). (**A**) Stacked bar plot showing relative sequencing read abundance of most abundant phyla. (**B**) Stacked bar plot showing relative sequencing read abundance of 20 most abundant genera. (**C**) Heatmap showing log_10_(relative abundance) *Vibrio* spp. Dendrogram shows k-means clustering of sampling location with respect to species relative abundance. Heatmap rows are ordered by abundance with dominant species on top. Red labels represent potential human pathogens.

SMS using DNA prepared from Sterivex concentrated and APW-enriched water samples generated an average of 18M (min = 13M; max = 21M) and 27M (min = 20M; max = 37M) paired reads across raw sequence read libraries, with a mean of 9M and 18M million unique paired reads, respectively. Following core gene metagenomic profiling, most reads were unclassified, while Sterivex concentrated and APW-enriched water samples generated on average 300K (min = 181K; max = 405K) and 1M (min = 720K; max = 1.4M) unique paired reads. Measures of alpha diversity (species richness, Shannon, and Simpson) are shown in Table 3. Overall, Sterivex-concentrated water samples revealed significantly higher alpha diversity compared to APW-enriched samples. Bacteria, archaea, fungi, protists, and viruses identified by DNA metagenomics are listed in Fig. 5, representing microbial taxa relative abundance (RA).

**TABLE 3:** Sequencing statistics and diversity indices

Method/sample	Accession no.	Richness	Shannon	Simpson
CC_S	SRR24799355	376.00	4.29	0.95
CK_S	SRR24799352	276.00	4.10	0.94
WBS_S	SRR24799349	397.77	4.25	0.94
CC1A_APW	SRR24799357	109.00	2.49	0.76
CC2B_APW	SRR24799356	118.00	2.50	0.76
CK1A_APW	SRR24799357	215.89	3.16	0.91
CK2A_APW	SRR24799353	174.00	3.12	0.90
WBS1A_APW	SRR24799351	153.00	2.76	0.87
WBS2A_APW	SRR24799350	153.00	2.93	0.88

*Proteobacteria* were most abundant, followed by *Actinobacter*, both detected in all samples. *Pseudoalteromonas* was the most abundant genus detected. Major differences were observed with respect to method, i.e., Sterivex concentrated vs APW enriched. For example, RA of *Proteobacteria* increased substantially after APW enrichment. Other differences were the dominance of *Bacteroidetes* in Sterivex-concentrated samples and the occurrence of *Fusobacteria* in APW-enriched samples. Genus level differences were also observed relative to method, with increased RA of *Vibrio* following enrichment (Fig. 5B). Vibrios were not detected in Sterivex-concentrated samples, whereas *Prochlorococcus*, *AVDB_g*, *Pelagibacter*, and *Pseudomonas* were dominant. APW-enriched samples revealed increased RA of *AB062844_g*, *Propionigenium*, and *Photobacterium*, with *Villovirus* as the dominant viral genus.

#### Pathogenic *Vibrio* spp.

Members of the genus *Vibrio* were detected after APW enrichment of the water samples and the *Vibrio* spp. are listed in Fig. 5C. Similar profiles were observed at all sample locations, and many members of the genus *Vibrio* were detected, including a number of species considered opportunistic and/or pathogenic. *V. alginolyticus*, *V. diabolicus*, and *V. parahaemolyticus* were the most abundant in all samples, followed by other members of the *V. harveyi* clade, including *V. harveyi*, *V. owensii*, *V. campbellii*, *V. natriegens*, and *V. rotiferianus*. Other pathogenic *Vibrio* spp. included *V. vulnificus*, *V. fluvialis*, and *V. furnissi* in all samples. *V. cholerae* group and *V. mimicus* were detected in CK and WBS samples, and *V. metschnikovii* and *V. cincinnatiensis* were detected only in a single sample from CK.

#### Detection of fungi, protists, and viruses

Whereas bacteria and archaea predominated, fungi, protozoa, and viruses were also present (Table 4). *Chlorophyta* was dominant in Sterivex samples but not detected in APW-enriched samples. Similarly, *Chrysochromulina ericina virus* was detected only in the Sterivex-concentrated water samples. The protist *Minutocellus polymorphus* and *Vibrio* phages, notably *Vibrio virus Kappa*, were dominant in APW-enriched water samples. It is worth noting *Vibrio virus CTXphi*, a phage encoding *V. cholerae* cholera toxin production, was detected in one sample from the CK station.

**TABLE 4:** Other taxa detected, listed as relative abundance (%)^*a*^

Phylum	Genus	Species	Cutthroat Clams			Clam Key Seafood			White Booth Seafood		
			CC_S	CC1A_APW	CC2B_APW	CK_S	CK1A_APW	CK2A_APW	WBS_S	WBS1A_APW	WBS2A_APW
**Viruses**											
Virus no name P	*Bracovirus*	*Cotesia glomerata bracovirus*	–	–	–	–	–	–	0.00	–	–
*Uroviricota*	*Igirivirus*	*Synechococcus T7-like virus* S-TIP37	–	–	–	–	–	–	0.10	–	–
*Uroviricota*	*Longwoodvirus*	*Vibrio virus* Kappa	–	–	–	–	0.05	0.02	–	0.04	0.18
*Phixviricota*	Virus no name G	*Chrysochromulina ericina virus*	0.11	–	–	0.70	–	–	0.93	–	–
*Hofneiviricota*	*Affertcholeramvirus*	*Vibrio virus* CTXphi	–	–	–	–	0.10	–	–	–	–
*Hofneiviricota*	*Fibrovirus*	*Vibrio virus* fs1	–	–	–	–	<0.01	–	–	–	–
*Hofneiviricota*	*Villovirus*	*Vibrio virus* Vf33	–	–	–	–	–	–	<0.01	–	–
**Protists**											
*Bacillariophyta*	*Minutocellus*	*Minutocellus polymorphus*	–	1.99	6.62	–	8.33	5.67	–	0.65	0.59
**Algae**											
*Chlorophyta*	*Nannochloris*	*Nannochloris* sp*.* X1	0.04	–	–	–	–	–	–	–	–
*Chlorophyta*	*Picochlorum*	*Picochlorum* sp*.* BH-2019	0.08	–	–	–	–	–	0.09	–	–
*Chlorophyta*	*Picochlorum*	*Picochlorum* sp*. ’soloecismus'*	0.05	–	–	0.06	–	–	0.05	–	–
*Chlorophyta*	*Picochlorum*	*Picochlorum* sp*.* SENEW3	0.10	–	–	2.75	–	–	4.05	–	–
*Chlorophyta*	*Picochlorum*	*Picochlorum oklahomense*	0.13	–	–	–	–	–	0.13	–	–

^*a*^ En dash “–” indicates not detected.

### Community resistome, virulome, and mobilome

**Fig 6:**
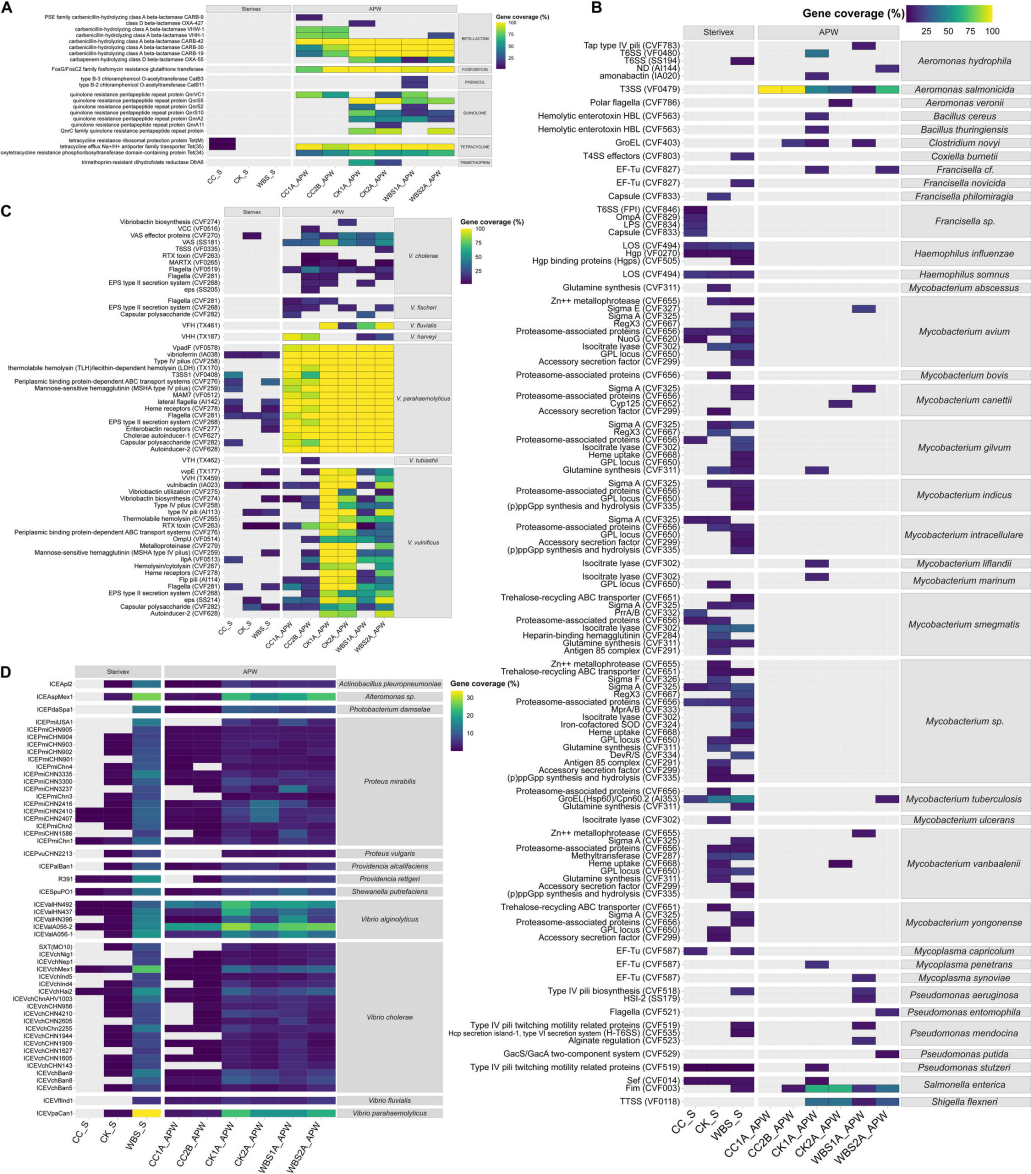
Microbiome community characteristics. Presence of (**A**) antimicrobial resistance genes using the NCBI National Database of Antibiotic Resistant Organisms (NDARO) database (66), (**B**) non-*Vibrio* virulence factors, and (**C**) *Vibrio* spp. Virulence factors using the Virulence Factor Database (61), and (**D**) integrative and conjugative elements using the Mobile Genetic Element Database (62). These were determined by mapping metagenomic sequencing reads to the respective databases using bowtie2 (67) with the “--very-sensitive” option and a Phred quality score threshold of 33.

Genes coding for antimicrobial resistance, virulence, and MGEs, namely, ICEs, were found at all stations (Fig. 6). Only a few ARGs were detected in Sterivex-concentrated samples, namely, tetracycline resistance, compared to APW enrichment (Fig. 6A). Genes encoding resistance to major antibiotic classes, including tetracycline, quinolone, fosfomycin, and beta-lactam, were detected at all stations, including trimethoprim resistance genes at CK and phenicol resistance genes at WBS.

VFs of non-*Vibrio* origin (Fig. 6B) were common in Sterivex-concentrated samples, and *Vibrio* spp. VFs (Fig. 6C) in APW-enriched samples. Of non-*Vibrio* VFs, those from *Mycobacterium* spp. were most common across all locations, notably in the Sterivex-concentrated samples. VFs of pathogenic *Vibrio* spp., namely, *V. cholerae*, *V. parahaemolyticus*, and *V. vulnificus*, were detected at all stations and at increased abundance following APW enrichment. *V. parahaemolyticus* associated VFs, namely, T3SS (type 1), were most common. *V. vulnificus* VFs included RTX toxin, VVH heme receptors, flagella, LOS, and autoinducers and *V. cholerae* VFs included RTX toxin, MARTX toxins, and T6SS.

Compared to Sterivex-concentrated samples, APW enrichment allowed detection of additional ICEs (Fig. 6D). ICEs originating in *V. cholerae*, *V. alginolyticus*, and *V. parahaemolyticus* were most prevalent, but ICEs originating in *Proteus mirabilis* were also common. Notably, two closely related ICEs, SXT and R391, respectively, associated with *V. cholerae* clinical isolates and *Providencia rettgeri* were detected at all stations and at increased abundance in the CK and WBS samples.

## DISCUSSION

Climate change is reported to have effects in most regions of the world and more so in coastal communities, notably through overall warming trends, altered precipitation patterns, and changes in jet streams and ocean currents. *Vibrio* spp. are naturally occurring in coastal waters globally, possess a high growth rate, and rapidly respond to environmental stimuli. The significant geographic expansion of pathogenic strains is correlated with impact on public health, i.e., increased number of infections caused by these pathogens (5–13). Archer et al. (13) recently showed a significant increase in *V. vulnificus* wound infections along the U.S. eastern seaboard. This observation is supported by data presented here, whereby both *V. vulnificus* and vibriosis (excluding cholera and *V. vulnificus*) infections reported in FL increased ca. fivefold and eightfold, respectively, between 1992 and 2022 (Fig. 1D).

It should be noted that climate change is also associated with increased frequency and intensity of severe weather events (68) and has the potential to alter the intensity and behavior of hurricanes, a notable example of which is Hurricane Ian, ranked the fifth strongest storm to hit the U.S. mainland (Fig. 1A), associated with a catastrophic storm surge and extreme rainfall. Hurricane Ian exemplified the more intense, slower-moving, and wetter signature commonly associated with a current generation of Atlantic storms influenced by climate warming (69). In the days following Ian, an increase in cases of vibriosis was recorded by the FL DOH, including 38 cases and 11 vibriosis-associated deaths attributed to the storm (case fatality rate of ca. 28.9%) (46). Between 28 September 2022 and 9 October 2022, *V. vulnificus* infections were most common, but *V. cholerae* non-O1/non-O139, *V. parahaemolyticus*, and *V. fluvialis* were also confirmed by FL DOH (46). Of the 11 deaths, nine were *V. vulnificus* and one *V. cholerae* non-O1/non-O139. Compared to Hurricane Irma, which made landfall in September 2017 and was responsible for six storm-related vibriosis cases documented in FL, 38 cases were reported after Ian. There was a lower storm surge during Irma compared to Ian (46), but in comparison with other vibriosis outbreaks caused by environmental catastrophes in the United States, the outbreak associated with Ian is notable because of the large number of hurricane-attributable cases occurring during a short period of time. In addition to storm surge, time series analysis for SST and chlorophyll (Fig. 1C) suggests some parameters of the coastal aquatic environment changed significantly during and after Ian, favoring the growth and proliferation of *Vibrio* spp.

Detection and characterization of pathogenic *Vibrio* spp. from the environment has been ongoing in the Chesapeake Bay since the 1960s (11, 16, 22, 23, 70–75). *Vibrio* spp. are native to and thrive in warm water with moderate salinity (10, 44), and pathogenic *Vibrio* spp. have been shown to be present in sediment during unfavorable environmental conditions, even when not detectable in water samples (11, 73). A recent culture-based investigation of *V. parahaemolyticus* and *V. vulnificus* in the Chesapeake Bay reaffirmed environmental predictors for these bacteria and documented their long-term increase and extended seasonality, notably during the fall (11). Specifically, critical environmental parameter thresholds were determined for SST (25°C), pH (8), DO (5 to 10 mg/L), and salinity (10–15 PPT) whereby increased to the maximum abundance of *Vibrio* spp. occurs. In areas with high salinity profiles, pathogenic vibrios are known to proliferate rapidly during heavy rainstorms that reduce the salinity and favor the growth of these bacteria, as demonstrated in the French Mediterranean (76), Chesapeake Bay (77), and Northern Gulf Coast (78). The salinity of the FL Gulf Coast is demonstrably higher than that of the Chesapeake Bay. However, considering the impact of Hurricane Ian’s heavy rainfall on salinity and other environmental parameters recorded 1 month post Ian along the FGC, including temperature, pH, and DO (Table 1), these values are within the range considered optimal for pathogenic vibrios to proliferate in the environment. In addition, chlorophyll serves as an important predictor of *Vibrio* abundance by indicating the density of phytoplankton populations, thus serving as an indicator of the subsequent proliferation of zooplankton, which feed on phytoplankton (5, 40, 79, 80). Here, it was observed that chlorophyll concentrations increased drastically during and after Hurricane Ian which would, by proxy, indicate increased zooplankton abundance with which *Vibrio* spp. are associated.

Cultures of *V. parahaemolyticus* and *V. vulnificus* isolated from water and oyster samples collected from the FGC subjected to WGS provided a core gene phylogeny analysis (Fig. 2) that matched previously proposed clades for *Vibrionaceae* (81), whereby each species formed coherent clusters within taxonomic subclades, in agreement with recent phylogenetic analysis (82). Species-specific core gene phylogeny was also done (Fig. 3), with subspecies clustering obtained for *V. vulnificus* (58) and *V. parahaemolyticus* (59). It was hypothesized that isolates from a single location would cluster with other strains from that location. However, distinct clustering patterns were not observed that linked strain phylogeny, source of isolation, or virulence capability, instead providing evidence for multiple clonal populations in circulation simultaneously. López-Pérez et al. (58) did a pangenome and phenotypic analysis that yielded two markedly different lifestyles with differentiated phylogenetic clusters, indicating commensal (C2) and bloomer (C1) ecotypes, with differences in carbohydrate utilization, defense systems, and chemotaxis. All *V. vulnificus* isolates from the study reported here fell into these two clusters. Based on the findings of Miller et al. (59), who performed WGS analysis to describe phylogenetic and biogeographic patterns of *V. parahaemolyticus* from North America, the FGC isolates in this study can be characterized as multiple sequence types, providing additional evidence indicating a hub of genetic variability of *V. parahaemolyticus* along the Gulf Coast.

Unlike clinical isolates, environmental *V. parahaemolyticus* strains generally do not encode primary VFs, such as thermostable direct hemolysin (*tdh*) and thermostable direct-related hemolysin (*trh*) (83–86). However, it has been estimated that up to ca. 27% of *V. parahaemolyticus* clinical isolates do not encode *tdh* and/or *trh* either (87), suggesting possible presence of other VFs. Furthermore, *tdh*/*trh* negative *V. parahaemolyticus* strains can cause severe infections in marine fish (88) and shrimp (89), with significant economic burden and aquaculture loss (90). *V. parahaemolyticus* isolated in this study did not carry *tdh* and/or *trh* but did have additional VFs, notably coding for the T3SS. ARGs were also detected in all *V. parahaemolyticus* strains isolated (Fig. 4). Interestingly, no single virulence gene has been identified that distinguishes pathogenic from non-pathogenic *V. vulnificus* (36). It is worth noting that RTX toxin, which promotes cytotoxicity and enhances survival of the bacterium during infection (91, 92), was detected in all *V. vulnificus* isolated in this study, and T6SS, a pilin apparatus contributing to biofilm formation, adherence to epithelial cells, and virulence (93), was detected in three of the FGC isolates.

*Vibrio* spp. have been shown to enter a protective state, namely, viable but nonculturable (VBNC), whereby the cells become metabolically dormant. That is, VBNC cells cannot be cultured using standard laboratory media yet are detectable using molecular genetic methods (94). Historically, detection of *Vibrio* spp. in the environment using culture methods had been challenging because of *Vibrio* spp. in the VBNC state, complicating detection and resulting in severe under representation of total *Vibrio* populations. VBNC cells have been shown to have increased resistance to thermal, low salinity, and acidic inactivation, suggesting this state plays a role in survival during adverse environmental conditions (95). Advances in molecular methods for microbial detection, identification, and characterization, notably next-generation sequencing and metagenomics, allow researchers to investigate more completely the mode of emergence and transmission of pathogenic agents that would have otherwise gone undetected (96). SMS is an effective molecular surveillance tool that allows bacterial, archaeal, viral, fungal, and protozoan microbiome community members to be identified and characterized. Demonstration of its value for microbial source tracking and wastewater surveillance linked to climate has been successful (97).

Traditional culture-based methods using selective media allowed successful recovery of *V. parahaemolyticus* and *V. vulnificus* from FGC samples. However, despite the evidence presented for multiple clonal populations in circulation, it should be noted that isolation-based studies may not accurately reflect the structure and complex dynamics of *Vibrio* populations (98). In contrast, culture-independent SMS of water samples concentrated using Sterivex filters was effective in profiling the microbiome, as shown for drinking water by Brumfield et al. (99). Here, SMS of Sterivex-concentrated water samples successfully profiled the community composition of environmental water samples but yielded an insufficient number of reads to be able to determine the total number of the *Vibrio* spp. with confidence. SMS of APW-enriched samples allowed the detection of many pathogenic species but not quantification because of enrichment. Nevertheless, *V. cholerae* and *V. fluvialis*, species of *Vibrio* confirmed to be the cause of deaths in FL linked to Hurricane Ian (46), were detected. Furthermore, WGS and SMS allowed characterization of the VFs, ARGs, and MGEs, along with multiple ARGs in the isolates of *Vibrio* spp. (Fig. 4A) and in the microbiomes (Fig. 6A).

### Challenges and future directions

Despite the broad application of PCR for use in diagnostics for infectious diseases and environmental pathogen detection, this technique yielded only limited information regarding the presence of *Vibrio* spp. in water and oyster samples in this study. Similarly, there was an inability to profile sequencing reads as members of the genus *Vibrio* via traditional SMS without enrichment (Fig. 5B). These observations were surprising considering the numerous *Vibrio* spp. detected following SMS with enrichment (Fig. 5C). However, as expected, SMS of Sterivex-concentrated samples showed greater alpha diversity than SMS with enrichment (Table 3). Culture-based enrichment allows the identification of diverse, rare elements in metagenomic sequencing (100), and enrichment using APW has been shown to be useful for amplification of *Vibrio* spp. (11, 101). However, it cannot be ignored that the process of enrichment creates a biased sample by nature (102). Hence, despite the ease of use of PCR and the high resolution of SMS, multiple methods should be considered for environmental surveillance.

Observations in this study were characterized using samples limited in number yet illustrative of the potential application of genetic analysis coupled with environmental data and remote sensing for public health by proactively detecting and characterizing environmental pathogens, notably *Vibrio* spp. The samples analyzed in this study were collected 1 month post Hurricane Ian and were not compared to samples from before the storm. Additional studies are needed to characterize fully the core microbiome and establish a comparative baseline of pathogenic agents and their public health significance. Work is in progress to compare the genomes of *Vibrio* spp. recovered from the environment, along with their VFs and ARGs, with clinical reports and cultures from patients. Future investigations would benefit from long-term sample collection and methods allowing for quantification, e.g., quantitative PCR (103) or DNA colony hybridization (11). In addition to more samples and other locations, different seasons of the year need to be studied. Such investigations would shed light on significant shifts in *Vibrio* numbers and the microbiome relative to changing environmental conditions as well as the incidence of other pathogenic agents.

### Conclusion

Climate conditions associated with the growth of and rapid response to environmental signals by *Vibrio* spp. make them a valuable microbial indicator of the impact of a changing global climate on public health (5–13). Between 1992 and 2022, a long-term increase in the number of confirmed *Vibrio* spp., namely, *V. vulnificus*, infections in the State of FL was documented (Fig. 1D), and the outbreak of vibriosis following Hurricane Ian serves as a traumatic example of the correlation of climate/weather processes and public health. In the study reported here, detection and characterization of pathogenic *Vibrio* spp. and the microbiome were achieved using a combination of PCR, culture, and advanced molecular sequencing. Observations in this study are for a limited number of samples collected 1 month after Hurricane Ian, yet microbial community profiles provide evidence for multiple pathogenic species, along with carriage of VFs, ARGs, and MGEs, in circulation in the FGC. In addition, this study provides useful information for future investigations, both for inhabitants of the FGC and globally, to evaluate trends in the incidence and genomic diversity of pathogenic *Vibrio* spp. and related microbial populations relative to environmental factors that enhance their growth and evolution in aquatic ecosystems. By employing satellite remote sensing, changes in the coastal aquatic environment can be coupled with WGS and metagenomic analysis to develop predictive risk models for *Vibrio* spp. and related pathogens. Such strategies will be critical as climate change accelerates over time.

## MATERIALS AND METHODS

### Environmental surveillance

The track, timeline, and windspeed data for Hurricane Ian were retrieved from the International Best Track Archive for Stewardship (IBTrACS) project (47). Chlorophyll and SST data were recovered for three locations, CC (26.569889,–82.135616), CK (26.546667,–82.079722), and WBS (26.36675,–82.03819), from the Moderate Resolution Imaging Spectroradiometer (MODIS) carried on the Terra satellite (104). MODIS data products are available at 4 × 4 km spatial resolution. A time series analysis was performed to determine the potential impact of anomalous weather events before (24 July 2022 to 23 September 2022), during (24 September 2022 to 30 September 2022), and after (1 October 2022 to 30 November 2022) Hurricane Ian. Anomalous percentages were calculated for SST and chlorophyll using the following equation: $Percentchange=\frac{x-LTavg}{LTavg}\times 100$ whereby “*x*” represents a given time period, i.e., before, during, or after, and “*LT avg*” represents mean of long-term data over 10 years for the same spatial resolution. Hence, anomaly percentage results in a positive or negative value, suggesting deviation for a given variable from the long-term mean. That is, negative anomalous percentages imply that a given variable decreased during 2022 with respect to the previous 10 years and positive values imply an increase.

### *Vibrio* spp. infections

Number of confirmed *Vibrio* spp. infections and associated deaths reported by the State of FL was retrieved from FL DOH (43, 45) and is presented as a number of cases for *V. vulnificus* and vibriosis (excluding cholera and *V. vulnificus*) between January 1992 and July 2023.

### Site description and sample collection

Methods employed for sample collection and processing have previously been described in detail (11). A summary of methods relative to this study is provided here, whereby samples were collected from three stations (CC, CK, and WBS) on 26 October 2022. At each station, water (7 L) was collected and stored in clean 3.7 L bottles. The bottles were rinsed three times with sample water from each site prior to collection. Roughly 12 oysters were collected from CK and WBS and stored in clean double zipper freezer bags. Oysters were not able to be collected from CC. All samples were transported to the laboratory in a cooler with ice, ensuring samples did not come in direct contact with the ice packs, and stored at 4°C until further processing. During each sampling event, water temperature, pH, DO, and salinity were measured 0.3 m below the surface and 0.3 m above the bottom using a handheld water probe (Eureka, Austin, TX).

### Sample processing

Samples were treated following methods outlined in the Bacteriological Analytical Manual for food sampling/preparation of sample homogenate (105) and *Vibrio* (106), as described previously (11). Notably, water samples were shaken vigorously 25 times in 30 cm arc in 7 s. From each station, 250 mL of water was concentrated four times (totaling 1 L) using syringe filtration with four 0.22 µm pore size Sterivex Filter Units (Millipore Sigma, MO). Oysters were rinsed and scrubbed under deionized water to remove debris from the shell and opened using a sterile shucking knife. Oyster tissue in equal part phosphate-buffered saline (pH 7.4) was homogenized in a sterile blender for 90 s. Filter units and homogenized oyster tissue (500 µL) were stored at −80°C in DNA/RNA Shield Stabilization Solution (ZymoResearch, CA). Following homogenization, subsequent enrichment steps were done within 15 min.

### APW enrichment

Samples were inoculated at various concentrations using APW (10% peptone, 1% NaCl [pH 8.5]). Briefly, three volumes of unfiltered water (1 L, 100 mL, and 10 mL) and homogenized oyster tissue (10 mL, 1 mL, and 100 µL) were resuspended in APW (10×), each in triplicate, and incubated overnight at 37°C with moderate aeration (orbit diameter 2.5 cm × 30 rpm). The following morning, an aliquot (500 µL) of each APW-enriched sample was stored at −80°C in DNA/RNA Shield Stabilization Solution (ZymoResearch, CA).

### Isolation of *Vibrio* spp*.*

A loopful of pellicle from each APW-enriched sample was subcultured on selective media, including *Vibrio* specific chromogenic agar (CHROMagar, Paris, France), thiosulfate citrate bile-salts sucrose (TCBS) agar (Oxoid, Ontario, Canada), and M190 *V. vulnificus* agar (107), and incubated overnight at 37°C. Presumptive *Vibrio* spp. colonies were purified on Luria-Bertani agar (Difco, NY) and maintained under standard bacteriological conditions for *Vibrio* spp*.* (108). Confirmation and identification of *Vibrio* spp. were done using established molecular assays, as outlined (see below).

### Preparation of genomic DNA

Genomic DNA was prepared from pure cultures grown under standard conditions in Luria-Bertani broth with aeration at 37°C overnight (16 h), using the ZymoBIOMICS DNA Miniprep Kit (ZymoResearch, CA). DNA extracts were further purified using DNA Clean and Concentrator Kit (ZymoResearch, CA), with a final elution volume of 80 µL.

### Polymerase chain reaction

PCR methods have previously been established for the detection of members of the genus *Vibrio* and species-specific markers. Amplified products were fractionated by electrophoresis through 1.5% (wt/vol) agarose gel along with a 100 bp molecular weight marker (HyperLadder, BioLine, Swedesboro, NJ) and visualized using SafeGLO Pre-Stain (BioLInk, San Francisco, CA). For quality control, a no template control (NTC) consisting of nuclease-free water and positive/negative controls was included with each reaction. Primers and respective controls used in this study are detailed in Table 2.

### Next-generation sequencing

Samples analyzed by next-generation sequencing included WGS of purified culture isolates identified by PCR as *V. vulnificus* (Vv*-toxR*^+^ and *vvhA*^+^; *n* = 12; Table S4) or *V. parahaemolyticus* (Vp*-toxR*^+^ and *tlh*^+^; *n* = 9; Table S5) and SMS of water samples (Table S6) from each location, both filter-concentrated (*n* = 3) and APW enriched (analyzed in duplicate; *n* = 6). Double-stranded DNA concentration was measured using the Qubit 3.0 fluorometer (ThermoFisher, MA). Sequencing libraries were prepared using NEBNext Ultra II FS Library Prep Kit for Illumina (New England Biolabs, MA) sequencing, using the HiSeq 4000 System (Illumina, CA) with 150 bp paired end reads. WGS was performed targeting >200 × genome coverage (>10 M paired reads), and SMS was done targeting >30 M paired reads. For quality control, an NTC and a sequencing standard, i.e., ZymoBIOMICS Community Standard (ZymoResearch, CA), were included in the sequencing run. Read quality was confirmed using FastQC (109). Adapter sequences were removed, and low-quality bases were trimmed using Trimmomatic (110). Processed reads were further analyzed for comparative genomics and metagenomic community profiling, as described below.

### Comparative genomics

Processed sequencing read libraries of purified culture isolates were assembled into contigs using the St. Petersburg genome assembler (SPAdes) (111), with options “--careful” and “--cov-cutoff auto” to reduce the number of mis-assemblies and remove low-coverage contigs. Small contigs (<500 bp) were discarded. Assembly statistics, completeness, and genome quality were assessed using the Quality Assessment Tool for Genome Assemblies (112) and CheckM (113). Draft genome assemblies were annotated using the Rapid Annotation Using Subsystem Technology tool kit (56) and characterized for carriage of antimicrobial drug-, biocide-, and metal-resistance determinants employing the Microbial Ecology Group Antimicrobial Resistance (MEGARes) database (60), VFs via the Virulence Factor Database (VFDB) (61), and MGE via the Mobile Genetic Element database (MGEdb) (62), using ABRicate (63) with identity and coverage thresholds set to 60%. MLSTs were predicted using the MLST software program (114) with the PubMLST database (115). Identified MLST profiles were submitted to the Public Databases for Molecular Typing and Microbial Gene Diversity (pubMLST) (115). Isolates identified as *V. parahaemolyticus* were subjected to serotyping by profiling serogroup-specific genes based on WGS data, using VPsero (116).

### *Vibrio* spp. phylogenetics

To evaluate the genomic relatedness of 21 purified culture isolates of this study with known *Vibrionaceae* strains, draft genome assemblies were compared with 77 representative *Vibrio* spp. genomes with established taxonomic lineages (Table S1), as defined previously (82). *Shewanella oneidensis* MR-1 (BV-BRC genome ID 211586.12) was used as an outgroup to root the tree. Phylogenetic trees were built using tools from the Bacterial and Viral Bioinformatics Resource Center (BV-BRC) (56). Briefly, Codon Tree method was used to select up to 1,000 genes from cross-genus protein families (PGfams), allowing for two gene duplications within a single genome and two genomes missing a member of a particular homology group. Coding DNA (amino acid sequences) from selected genes was analyzed using the Randomized Axelerated Maximum Likelihood (RAxML) algorithm with automatic model selection (57) to identify the best model for protein alignment. The resulting codon trees were viewed through the Phylogenetic Tree Viewer in the BV-BRC software suite (56).

Similar methods were used to further classify isolates from FL within existing *V. vulnificus* and *V. parahaemolyticus* clonal populations. Briefly, 12 isolates identified as *V. vulnificus* were analyzed along with 88 representative strains (Table S2) (58) using the BV-BRC Codon Tree method, as mentioned previously, but up to five gene duplications and genome deletions were allowed. Similarly, the nine isolates identified as *V. parahaemolyticus* were analyzed along with 91 representative strains (Table S3) (59), allowing for 10 gene duplications and genome deletions. The resulting codon trees were visualized using the Interactive Tree of Life (117).

### Metagenomic community profiling

Genomic sequences in public repositories can have a diverse range of genome statistics, e.g., reference size, number of contigs, assembly status (i.e., complete, chromosome, scaffold, contig), N50 values, etc., which may introduce a bias toward higher quality and complete genomes, thereby making abundance quantification unreliable. To circumvent this issue, metagenomic community profiling was done using the up-to-date bacterial core gene (UBCG) algorithm (64) with the EzBioCloud database (65). When extracting UBCG sequences, all references end up being represented by the same number of genes, and their sequence sizes are nearly identical, making detection and abundance estimation more reliable. The 92 core genes (bacteria and archaea) currently hosted by the UBCG algorithm were extracted from the EzBioCloud database to create a core gene database for metagenomic profiling. First, the potential presence of bacterial and archaeal species was surveyed for each metagenomic sample read using Kraken2 (118) and a pre-built core gene database (64) containing k-mers (*k* = 35) of reference genomes obtained from the EzBioCloud database (65). Fungi, protists, and viral strains (full genomes) were extracted from the NCBI RefSeq database (119) and added to the core gene database. After acquiring a list of candidate species, a custom bowtie2 (67) database was built from species detected during the Kraken2 (118) analysis. Sample reads were mapped against the bowtie2 database using the “--very-sensitive” option, and a Phred quality score threshold of 33. SAMtools (120) was used to convert and sort the resulting bam file. Coverage of the mapped reads against the bam file was obtained using BEDtools (121). To avoid false positive calls, reads mapping to a given species were quantified only if the total coverage of their core genes (bacteria and archaea) or genome (fungi, protists, and virus) was at least 25%. Species abundance was calculated using the total number of reads counted, and normalized species abundance was calculated using the total length of the respective reference sequence. Measures of alpha diversity (species richness, Shannon, and Simpson) were calculated using the EzBioCloud software platform (65), as described previously (122). Briefly, Species richness was defined using the abundance-based coverage estimator algorithm (123). Shannon entropy of counts was calculated based on the description given in the Species Diversity and Richness manual (124). However, log base 2 (log_2_) was used as default instead of the natural logarithm (log_e_). Simpson’s index was defined as “1 – *Dominance*” whereby dominance represents the probability of selecting two individuals from the same species, with replacement (125).

ARG, VF, and MGE profiles were produced using separate pre-built bowtie2 (67) reference gene databases composed of the NCBI National Database of Antibiotic Resistant Organisms (NDARO) database (66), VFDB (61), or MGEdb (62). Metagenomic reads were mapped against respective databases using bowtie2, as previously mentioned. For each gene detected, depth and coverage were calculated using mpileup script from the SAMtools software package (120).

### Software

Statistical analysis was done using R v.4.2 (126), EzBioCloud (65), and BV-BRC (56). Figures were generated using ggplot2 (127), iTOL (117), and Phylosmith (128).

## Data Availability

Sequencing data generated for all samples included in this study are deposited in the National Center for Biotechnology Information Sequence Read Archive database under BioProject ID PRJNA978582. Accession numbers and MLST profiles for draft genome assemblies of *V. vulnificus* (Table S4) and *V. parahaemolyticus* (Table S5) and microbiome sample sequencing read libraries (Table S6) are provided in the supplementary information.

## SUPPLEMENTAL MATERIAL

Supporting information mbio.01476-23-s0001.xlsx Tables S1–S6.

ASM does not own the copyrights to Supplemental Material that may be linked to, or accessed through, an article. The authors have granted ASM a non-exclusive, world-wide license to publish the Supplemental Material files. Please contact the corresponding author directly for reuse.
